# NAD+ Precursors: A Physiological Reboot?

**DOI:** 10.3390/nu15204479

**Published:** 2023-10-23

**Authors:** Julia Niño-Narvión, Mercedes Camacho, Josep Julve

**Affiliations:** 1 Institut d’Investigació Biomèdica Sant Pau (IIB Sant Pau), 08041 Barcelona, Spain; julianng99@gmail.com; 2 Department of Biochemistry and Molecular Biology B and Immunology, Faculty of Medicine, University of Murcia (UMU), 30120 Murcia, Spain; 3 CIBER of Diabetes and Related Metabolic Diseases, Instituto de Salud Carlos III, 28029 Madrid, Spain

In this Editorial, we comment on a series of recent articles featured in the Special Issue “Emerging Benefits of Vitamin B3 Derivatives on Aging, Health and Disease: From Basic Research to Translational Applications” in *Nutrients*. These articles address the relevant aspects of nicotinamide adenine dinucleotide (NAD+) depletion across a wide spectrum of pathological conditions, including metabolic, neurological, and age-related disorders, as well as the potential health benefits of NAD+-raising approaches.

Beyond the well-established role of NAD+ in redox reactions, this key molecule also acts as an essential co-substrate for the activity of a wide range of regulatory proteins. Some of these proteins are involved in modulating pivotal cellular processes that are fundamental to cell physiology, such as NAD+-dependent histone deacetylases, also defined as sirtuins, polyADP-ribose polymerases (PARPs), and cyclic ADP-ribose synthases, among others.

Accumulating evidence supports the notion that NAD+ metabolism is commonly distorted in different pathophysiological scenarios and that its restoration might be considered a plausible therapeutic strategy to mitigate the progression of adverse outcomes directly related to NAD+ deficient states [1,2]. Over the past decade, specific dietary manipulations of NAD+ using natural forms of vitamin B3 [3]—namely nicotinic acid, nicotinamide, nicotinamide mononucleotide, and nicotinamide riboside (NR)—have been shown efficient in reversing detrimental outcomes of chronic metabolic/inflammatory diseases and age-related disorders in animal models. Consequently, the development of NAD+-increasing therapies stands as one of the most exciting challenges when it comes to improving human health and lifespan [4,5,6]. However, translating the promising effects that have been observed in experimental models into clinical benefits has only yielded modest results. At least partly, the latter could be attributed to several factors, such as the extent of NAD+ depletion, the efficacy of NAD+ repletion, or both, which could be blunting the reversal of the adverse phenotype by the NAD+-raising approach, thereby limiting data interpretation. 

In contrast to the extent of NAD+ depletion, which is closely tied to the presence and severity of specific disease states, the efficacy of NAD+ repletion may be more accurately controlled. A notable example is NR, one of the widely studied NAD+ precursors. Due to its unique characteristics, NR is especially more prone to degradation in the gastrointestinal tract. Extensive research has therefore focused on generating different forms of this nicotinamide derivative, incorporating differences with various chemical groups into its molecular backbone to overcome this limitation [7]. Despite this, NR bioavailability for NAD+ synthesis in tissues also hinges on the activity of nicotinamide riboside kinases. These enzymes are predominantly found in the liver and kidney but are poorly expressed in other organs, potentially hampering the biological action of NR. In this context, a derivative of NR-dihydronicotinamide riboside (NRH) has recently emerged as a new NAD+ precursor [8,9], displaying a more potent NAD+ raising capacity than other NAD+ precursors.

In this context, Ciarlo E. et al. [10] published a contribution to NAD+ metabolism in cultured hepatocytes by another molecular player, dihydronico-tinic acid riboside (NARH). In their research, the authors demonstrated that NARH can also act as a NAD+ precursor. Indeed, in combination with NR, this new NR derivative enhances the overproduction of intracellular NAD+ in cultured hepatocytes and induces the synthesis of NRH. Although further research is needed to understand the therapeutic potential of NAD+ precursor combinations, including NARH and its clinical use, this kind of study opens new venues in the investigation of the physiological impact of novel NAD+-raising therapies on global NAD+ homeostasis.

Inflammation is one of the most common pathophysiological mechanisms that frequently accompanies the course of metabolic, neurodegenerative, and age-related disorders. In this Special Issue, Sharma C. et al. [11] extensively reviewed the favorable influence of NR supplementation in different experimental settings. Notably, in their review, the authors exhaustively described the anti-neuroinflammatory effect of NR in different animal models and its positive influence on neurological disorders. Of particular interest was the mechanism of action of NR-mediated protection against neuroinflammation, which was elegantly dissected in a specific mouse model of neuroinflammation, the Gulf War illness (GWI) mice. Interestingly, these mice present depleted brain NAD+ levels along with neuroinflammation [12]. Conceivably, an increased abundance of proinflammatory cytokines was detected in the brains of GWI mice, whereas NAD+ repletion by NR in treated GWI mice abrogated neuroinflammation [12]. In contrast to other studies, the anti-inflammatory effect of NR was, at least in part, explained by an induced deacetylation of the NFκB isoform p65 (at amino acid residue K310), which is the nuclear and the active form of this master regulator of inflammation. This finding coincides with another recent study published in this Special Issue, which documented the efficacy of another NAD+ precursor (nicotinamide) in ameliorating brain inflammation via NAD+-dependent deacetylation mechanisms of the same NFκB isoform (p65) in a different mouse model of neuropathy, i.e., diabetic neuropathy [13]. In this study, the favorable influence of nicotinamide on brain inflammation was directly linked to concomitant NAD+ elevations in the brains of nicotinamide-treated diabetic mice. Additionally, microglial activation was attenuated upon nicotinamide administration. Together, these studies provide support for the strategy of reversing neuroinflammation with NAD+ repletion therapy.

The burden of another severe syndrome caused by SARS-CoV-2 infection and associated with acute respiratory syndrome has also been related to disturbances in NAD+ metabolism. Supporting this, NAD+ depletion is a common feature in certain viral infections [14,15]. Sharma C. et al. [11] also linked the NAD+ depletion in target SARS-CoV-2-infected tissues to the overexpression of PARPs, a class of NAD+-consuming enzymes [16], and the potential benefits of emerging NAD+-increasing approaches as relevant adjuvant therapy to alleviate the severity of COVID-19. In this regard, clinical trials investigating the impact of NAD+-replenishment-based therapies on COVID-19 manifestations are currently underway. This therapy offers no side effects and is low-cost, and although new data from these clinical trials are still awaited, current research suggests that it could be useful for treating SARS-CoV-2 infection.

Beyond its role as a NAD+ precursor, Sharma C. et al. also mentioned that NR has been proposed to potentially act as an inhibitor of SARS-CoV-2 RNA-dependent RNA polymerase, a key enzyme in viral genome replication and gene transcription. In support of this, it has been proposed that NR may exhibit antiviral activity due to its molecular similarity to nucleoside inhibitors, which are a class of molecules that inhibit the activity of the abovementioned polymerase. Together, this evidence might support the hypothesis that NR’s efficacy against COVID-19 is directly influenced by either its NAD+-increasing capability, structural similarity to nucleoside inhibitors, or both.

Furthermore, NAD+ precursors show promise in maintaining homeostasis in systems/organs beyond the nervous system, such as the gut barrier. In this regard, Niño-Narvión J. et al. [17] produced an excellent comprehensive exploration of the contribution of vitamin B3 derivatives to gut health, developing the concept that there is a close relationship between NAD+ metabolome and gut inflammation. Despite the fact that current evidence supports the notion that NAD+ deficiency enhances gut inflammation and that this is frequently accompanied by concomitant changes in intestinal dysbiosis, emerging research suggests the positive influence of NAD+-increasing strategies on intestinal microbiota composition; however, whether such favorable, NAD+-mediated changes in intestinal microbiota could protect against gut inflammation and leakage has been so far poorly explored. In this respect, Niño-Narvión J. et al. [17] launched and investigated the hypothesis that administering NAD+ precursors could be beneficial in protecting the gut against inflammation in various pathophysiological scenarios by modulating the intestinal microbiota. Overall, these findings pave the way for future investigations.

In conclusion, the landscape of NAD+ precursors and their therapeutic potential is rapidly unfolding. The collection of articles featured in this Special Issue provides a comprehensive exploration of the critical role NAD+ plays in health and disease across a range of different pathophysiological contexts. The intricate relationship between NAD+ deficiency and adverse outcomes, either metabolic or inflammatory, is thus becoming more evident. In this context, the potential health benefits of NAD+-raising strategies, ranging from countering inflammation, i.e., neuroinflammation, and mitigating the severity of viral infections to influencing gut health, showcase the versatility of NAD+ restoration. Accumulating experimental research has revealed NAD+-dependent sirtuin signaling as being one of the main effectors of NAD+-increasing therapies. Nonetheless, while experimental models have shown promise, translating these findings into clinical practice poses challenges. Optimizing the efficacy of NAD+ repletion, particularly in the face of varying degrees of depletion, remains a pivotal concern. However, advancements in the development of novel NAD+ precursors and derivatives offer hope for more targeted and effective interventions. In summary, the pursuit of NAD+-replenishment-based therapies continues to shape the landscape of modern medicine. In this context, rigorous translational and clinical research efforts are essential to bridge the gap between experimental promise and clinical impact. By unraveling the intricacies of NAD+ metabolism and its relationship to various health conditions, we move ever closer to realizing the potential of NAD+ precursors as powerful tools for enhancing human health and longevity.
